# Controlling Normal Stiffness in Droplet-Based Linear Bearings

**DOI:** 10.3390/mi9100525

**Published:** 2018-10-17

**Authors:** Qi Ni, Nathan Crane

**Affiliations:** 1 Mechanical Engineering Department, University of South Florida, Tampa, FL 33620, USA; qni@mail.usf.edu; 2 Mechanical Engineering Department, Brigham Young University, Provo, UT 84602, USA

**Keywords:** surface tension, capillary, bearing, wetting

## Abstract

While capillary forces are negligible relative to gravity at the macroscale, they provide adequate force to effectively manipulate millimeter to micro meter objects. The fluidic actuation can be accomplished using droplets that also act as bearings. While rotary droplet bearings have been previously demonstrated, this paper addresses the positioning accuracy of a droplet-based bearing consisting of a droplet between a moving plate and a stationary substrate with constrained wetting region under a normal load. Key wetting cases are analyzed using both closed form analytical approximations and numerical simulations. The vertical force and stiffness characteristics are analyzed in relation to the wetting boundaries of the supporting surface. Case studies of different wetting boundaries are presented and summarized. Design strategies are presented for maximizing load carrying capability and stiffness. These results show that controlled wetting and opposing droplet configurations can create much higher stiffness fluidic bearings than simple droplets.

## 1. Introduction

**Figure 1:**
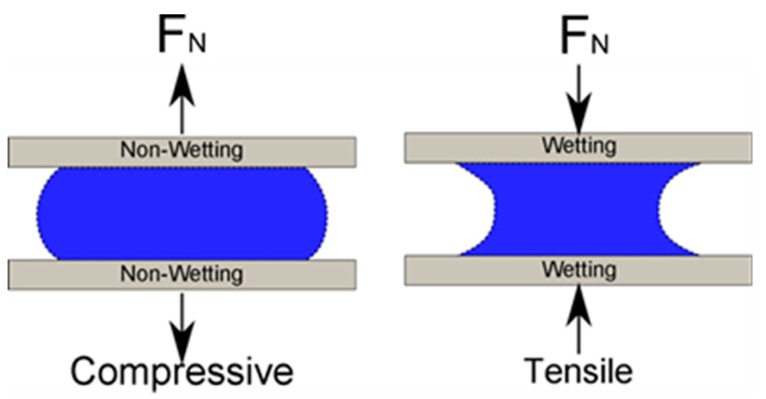
Tensile and compressive forces from a capillary bridge formed by two parallel surfaces.

Due to scaling relations, capillary force becomes dominant as the length scale decreases below the millimeter scale and can dominate from micrometer to millimeter scales [1]. The relatively large magnitude of the capillary force has been exploited to perform mechanical operations such as grasp and release [2,3], out-of-plane self-assembly [4], and vertical actuation via electrowetting [5]. A common gripper configuration utilizes the tensile force exerted by a capillary bridge between two wetting surfaces. When the surfaces are non-wetting (contact angle is greater than 90°), a repelling force is generated when the two surfaces are close together [6,7] as illustrated in Figure 1. Electrowetting, an apparent change in surface energy or contact angle due to electrostatic forces acting on a surface, can be used to modulate these forces [8,9].

A new class of bearing based on the capillary bridge was proposed by several groups and rotating micromachines were demonstrated in references [10,11,12,13,14]. The bearing surfaces were patterned by either surface coating and/or surface texture to control the location and shape of the droplet/surface contact. Single droplets, multiple drops, and fluid rings have been used to support vertical loads by surface tension. The advantages of this type of bearing are low friction, wear resistance and self-centering [10]. In addition, external force/torque can be applied either by external field to the rotating rotor [13] or through the droplet [12,14] which could further reduce device size.

The same principle could be further extended to use droplets to support vertical loads in linear translational motion. With electrowetting actuation, the droplets can serve as both bearing and actuator. Moon and Kim demonstrated this concept by using electrowetting to move droplets which carried a solid platform for biochemical analysis [15]. In their demonstration, a voltage was applied to the electrodes on the actuation substrate which induced a shape change within the droplets. This propelled droplet translation and the droplets transferred the force to the solid platform. The force is transferred to the moving element through the surface tension. The droplet interface on the actuated object is constrained, by a defined wetting pattern created by a local coating and/or surface texture. For an aqueous actuation droplet, the surface inside the wetting region is hydrophilic and outside it is hydrophobic. Motion accuracy depends on maintaining a constant spatial relationship between the droplet/solid surfaces of the droplet. For integrated EW-actuated linear motion, the electrowetting effect actuates the droplet which is supporting the plate, so the bottom surface must be hydrophobic.

For linear bearings, the stiffness and load carrying capacity are critical parameters. The vertical stiffness determines the precision of the bearing in the transverse direction under varying loads. For rotational bearings, it was noted in reference [10] that the maximum speed of the rotor was highly dependent on the thickness of the droplet due to the viscosity of the fluid. It is important to optimize the load and the fluid volume which have large influence on the resulting gap height. While valuable prior work has been done on the force of capillary bridges [5,16] and their dynamics [17], these do not consider their use as linear bearings. This paper develops static force and stiffness relations for circular droplets supporting normal loads between two parallel plates. These provide critical information about the load carrying capability of droplet-based bearings and the positioning accuracy at slow speeds. Analytical approximations are compared to numerical simulation of surface forces under varying surface constraints (wetting boundary and/or geometrical boundary). Depending on the wetting properties of the surfaces, different stiffness and load capability can be utilized for droplet based bearings. Based on these results, design strategies for droplet linear bearings are proposed. This work can serve as a design guideline to implement droplet based linear bearings in electrowetting driven application, as well as in designing droplet-based rotating machines.

## 2. Materials and Methods

In its simplest form, a droplet-based bearing consists of a capillary bridge formed between two parallel surfaces. The interfacial tensions act at the triple point where the liquid, solid, and ambient fluid meet. The angle between the liquid and solid is the contact angle. Lambert and co-workers have shown that both the Laplace approach and interfacial energy approach are equivalent [18]. In here, we will take the Laplace approach. Given the condition that the characteristic dimension is much less than the capillary length $\sqrt{\frac{\gamma }{\rho g}}$) (where *γ* is the surface tension, ρ the density difference between the droplet and the ambient, and *g* the acceleration due to gravity), the effect of gravity on the fluid can be neglected. For water in an air ambient, the capillary length is approximately 2.7 mm.

**Figure 2:**
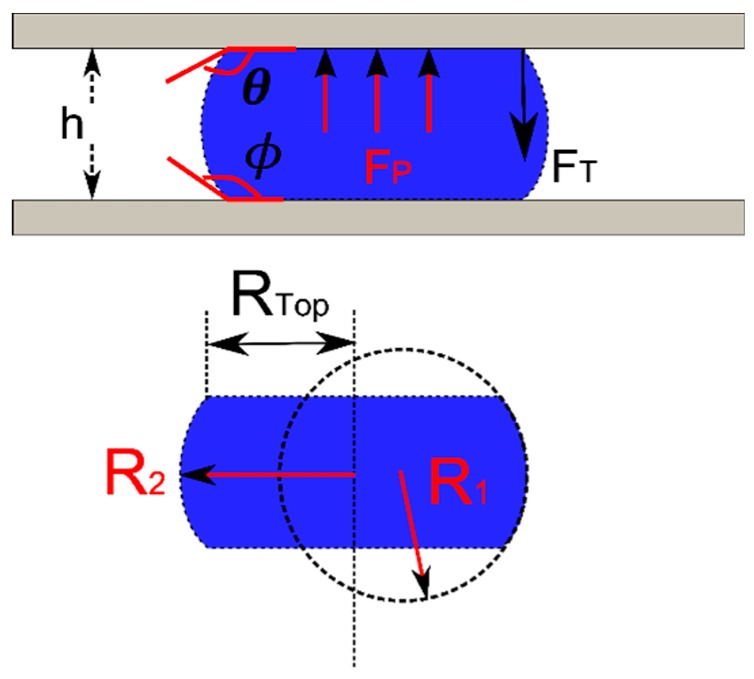
Capillary bridge between two parallel surfaces. When the top and bottom contact angles (*θ* and *Φ*) are greater than 90°, the pressure force (*F_p_*) pushes the top plate upwards and the surface tension force (*F_T_*) acts downwards to pull the plates together. The two principle radii of curvature (*R*_1_ and *R*_2_) can be are used to calculate the pressure difference across the interface and *R_Top_* is the contact radius of the droplet to the top plate.

As shown in Figure 2, the bottom surface is fixed and the separation distance (*h*) is known. The forces acting on the top plate are the surface tension force and the pressure force. The vertical component of the surface tension (*F_T_*) acting on the top plate is found by integrating around the interface. For the case of a circular droplet with constant wetting angle:

(1) $$F_{T}=2\pi R_{top}\times \gamma sin\left(\theta \right)\;$$

where *γ* is the interfacial tension of the liquid-ambient, *R_top_* is the radius of the contact area and *θ* is the contact angle of the liquid and top plate. The pressure force, which originates from the pressure jump across the liquid/ambient interface can be calculated using the Laplace pressure equation:

(2) $$\;F_{P}=\pi R_{top}^{2}\times \Delta P=\pi R_{top}^{2}\times \gamma \left(\frac{1}{R_{1}}+\frac{1}{R_{2}}\right)\;$$

where, *R*_1_ and *R*_2_ are the principle radii of the curved interface. Giving the separation distance (*h*) between the plates, one principle radius can be found by geometry:

(3) $$\;h=-R_{1}\times \left(cos\left(\theta \right)+cos\left(\phi \right)\right)\;$$

where the contact angle with the moving and stationary plates are *θ* and ϕ, respectively. The pressure can be either positive or negative depending on the contact angles. When both angles are larger than 90°, the pressure is positive, which acts to push the plates apart. When both angles are less than 90°, the resulting negative pressure force will pull the plates together. If the radius of the capillary bridge is much larger than the separation distance (*R*_2_ >> *h*), the second principle radius can be approximated by the radius of the plate contact (*R*_2_ = *R_top_*). The top radius is used as it will be assumed to be prescribed while the other may vary with load. Then the normal force (*F_N_*) on the top plate is as follows:

(4) $$\begin{array}{c} \;F_{N}=F_{P}-F_{T}=\pi R_{top}^{2}\times \gamma \left(\frac{1}{R_{1}}+\frac{1}{R_{2}}\right)-2\pi R_{top}\times \gamma sin\left(\theta \right)\\ =\pi R_{top}\gamma \times \left[-\frac{R_{top}}{h}\left(cos\left(\theta \right)+cos\left(\phi \right)\right)+1-2sin\left(\theta \right)\;\right]\; \end{array}$$

The equation is nondimensionalized by normalizing the height ($\bar{h}=h/2R_{top}$) with the diameter of the drop (*D =* 2*R_top_*) to get the following:

(5) $$\;\frac{F_{N}}{\pi D_{top}\gamma }=\frac{-cos\left(\theta \right)-\;cos\left(\phi \right)}{4\times \bar{h}}+0.5-sin\left(\theta \right)\;$$

For droplet-based bearings, the contact radius/diameter of the top surface can be controlled by either surface coating or roughness so that inside of the wetting region, the surface is hydrophilic. Outside of the region, the surface is hydrophobic with a contact angle greater than 90°. The key quantities of interest are the normal load capability and stiffness of droplets with known bottom contact angle, plate spacing, and droplet volume. In practice, the actual contact angles may not be known a priori at the boundary. If the contact line is at the boundary between two regions with different effective contact angles, the contact angle can assume any value between the interior and exterior wetting angles without moving the contact line. These relationships will be used to develop estimates of the forces, but are not suitable for calculating actual fluid reactions in many cases of practical interest.

**Figure 3:**
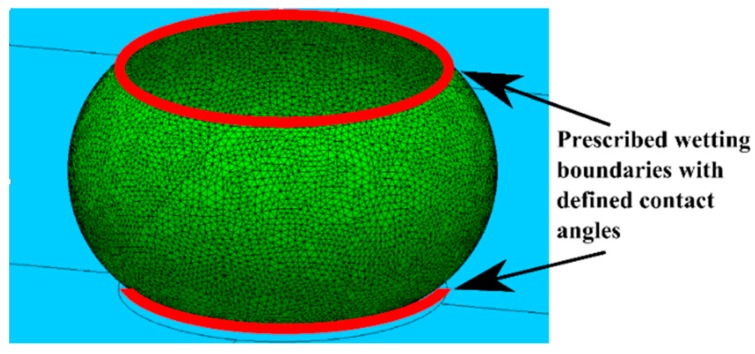
An example of the simulation boundaries. Inside the red lines, the surface energy is defined by low contact angle (10°). Outside the red lines, high surface energy is defined by large contact angle (110°–165°).

The reaction forces in more general cases are calculated numerically using software such as Surface Evolver [19]. Surface Evolver calculates the total system energy by discretizing the surface and summing a series of surface and line integrals. The equilibrium shape is found by gradient-based energy minimization. In the simulation, a droplet with defined volume and contact angle condition on the top and bottom plate was constrained between two planes that represented the substrate (bottom) and moving plate (top). An example of the simulation boundaries is presented in Figure 3. The effect of gravity was not included since the desired operating range of the droplet based bearing is substantially less than the capillary length.

Once the equilibrium shape of the surface was found, a small displacement in the z direction (50 µm) was applied and the energy change was calculated. The normal force was found using the principle of virtual work (*F_z_* = *dE*/*dz*) [20,21]. Results of surface evolver calculations are compared to the limiting closed form approximations (Equation (5)) for relevant wetting boundary conditions below. These numerical predictions of normal forces for a range of wetting conditions and droplet/gap ratios will be compared to simplified assumptions based on the analytical solutions. These results will be used to identify promising configurations for high stiffness fluidic bearings.

## 3. Results

An effective fluid bearing must be able to support the normal loads applied and should also have a high stiffness to minimize the displacement caused by changes in applied forces. While the force and stiffness are linearly dependent on surface tension, the droplet diameter, the substrate/plate gap, and contact line constraints will nonlinearly impact the performance of the fluid bearing. This paper considers the impact of the droplet aspect ratio (gap height/diameter) for three different wetting arrangements in a simple fluid bearing for different contact line constraint conditions.

### 3.1. Type 1—Uniform Wetting

For axisymmetric wetting, without wetting boundaries, the contact angles of the top and bottom are the same (*θ* = *Φ*), Equation (5) reduces to:

(6) $$\;\frac{F_{N}}{\pi D_{top}\gamma }=\frac{-cos\left(\theta \right)}{2\times \bar{h}}+0.5-sin\left(\theta \right)\;\;$$

This case is used as a bench mark for the Surface Evolver simulation. The surface tension for all types was fixed at 0.072 N/m. The variables used are listed in Table 1.

**Figure 4:**
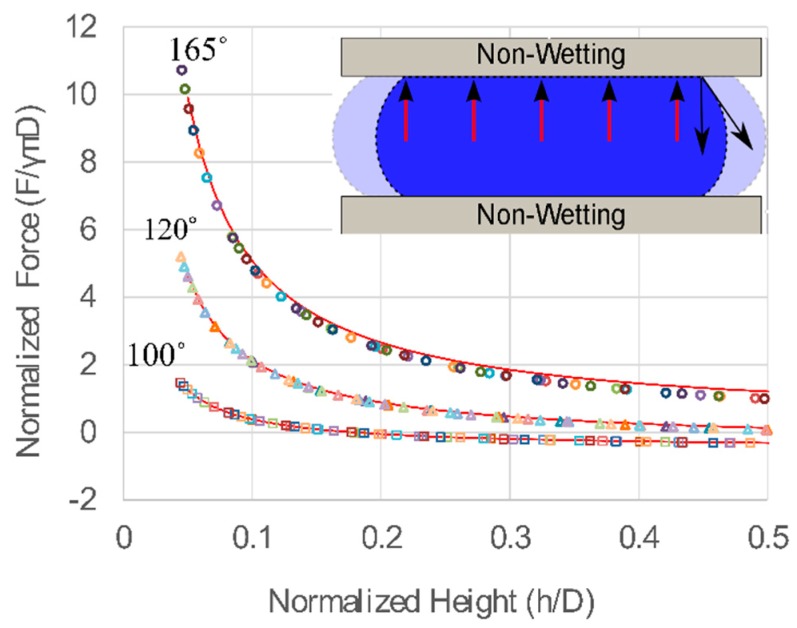
Normalized force vs. normalized height, Type 1. For a symmetric capillary bridge on a non-wetting surface, the contact angles are the same top and bottom, circular, triangle and square marks are the simulation results for contact angles of 165°, 120° and 100°, respectively. Each data set includes simulation results for 10 droplet volumes used (5–50 µL). The solid lines are the calculated values from Equation (6).

The normal force at each condition was extracted and the resulting forces and the top contact diameter were extracted from the models and the normalized values are summarized in Figure 4. When normalized, the force for a given contact angle collapses to a single curve for all droplet volumes. The simulation data (markers) agrees well with Equation (6) predictions (solid lines) for small values of aspect ratio for all contact angles. Good agreement is seen at larger aspect ratios and for contact angles closer to 90° where the assumptions in Equation (6) are most accurate. For large contact angles (165°), Equation (6) over predicts compared to the simulation, especially at larger aspect ratios. The error arises from using the contact radius as a principle radius of curvature and assuming cylindrical droplet shape for calculating the diameter. However, Equation (6) gives a good approximation of forces over the design region of greatest interest (small aspect ratios) for all contact angles evaluated.

At large aspect ratios, the droplet force is relatively insensitive to the aspect ratio, but this equates to a low stiffness. However, when the aspect ratio is less than 0.1 the slope is much higher—creating a stiffer bearing in which position is less sensitive to applied loads. Alternatively, the higher normal force could allow the bearing diameter to be reduced for the same force/stiffness capacity.

**Figure 5:**
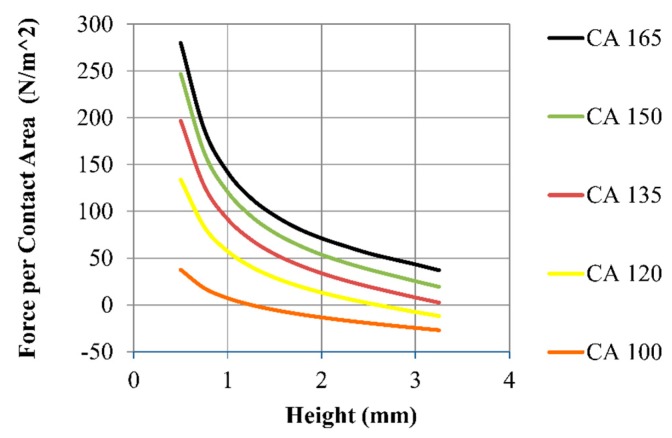
Force per unit area calculated from Equation (6). Each line indicates the different contact angles used for the top and bottom surfaces. The droplet’s volume was fixed at 50 µL.

For hydrophobic surfaces, the surface tension forces pull the moving plate toward the substrate—opposite the pressure force. As the contact angle increases, the surface tension has a smaller normal components and acts to increase the pressure increases resulting in much larger normal forces. Figure 5 shows the calculated force per unit contact area on the top plate with a 50 µL droplet at various contact angles from Equation (6). As the contact angles increase from 100° to 165°, the force increases by a factor of 5 or more (see Figure 5). Higher contact angles and/or smaller aspect ratios should be used to support larger vertical loads.

### 3.2. Type 2—Defined Wetting Region

**Figure 6:**
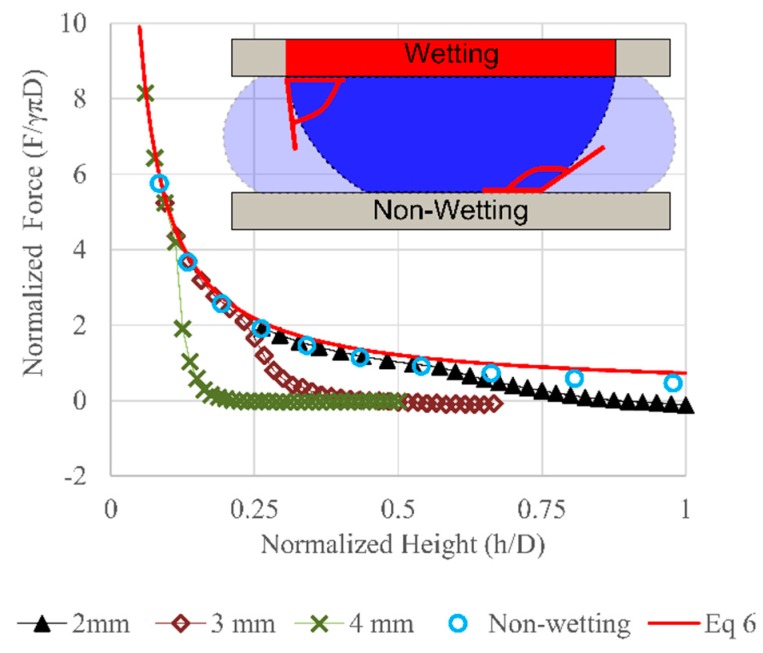
Normalized force vs. Normalized height with Type 2 wetting. The force were normalized by the product of the surface tension, π and the top contact diameter. The circles are the simulation result from Type 1 (symmetric non-wetting, contact angle: 165°), the line is the prediction from Equation (6). The cross, square, and triangle marks are the simulation results for different wetting radius (4, 3, 2 mm) when the droplet’s volume was fixed at 50 µL. The inset shows the wetting region on the top plate. As the gap height betweent the plate decrease, the droplet sperad out to the non-wetting region.

In a practical bearing, the droplet contact line must be constrained relative to one of the plates while sliding across the other. This could be done by defining a specific wetting region on one plate while the other plate remains hydrophobic—breaking the symmetry of the droplet wetting. This non-symmetric wetting was simulated in Surface Evolver using a hydrophilic circular region on the top (moving) plate (contact angle = 10°) with different wetting radius (*R_top_* = 2, 3, and 4 mm). The remainder of the top surface and the entire bottom were non-wetting (contact angle = 165°). Droplet volume was fixed at 50 µL. The vertical forces were extracted and then normalized by the top contact diameter and compared to Type 1 performance for various droplet heights. The result is presented in Figure 6.

At small heights, the force follows the Type 1 curve until the wetting region on the top plate reaches the edge of the wetting region. As aspect ratio continues to increase (height grows), the force drops dramatically relative to the Type 1 case as the bottom plate contact area decreases while the top contact line remains stationary. While the lower normal force in this region is undesirable, the increased stiffness in the transition region is favorable for reducing variation in plate positioning with applied normal forces. For a given wetting area, this also allows the bearing to operate at a higher gap height with favorable stiffness. Larger gaps and smaller aspect ratios should reduce the drag introduced by the fluids in a linear actuator.

### 3.3. Type 3—Constrained Top Wetting

**Figure 7:**
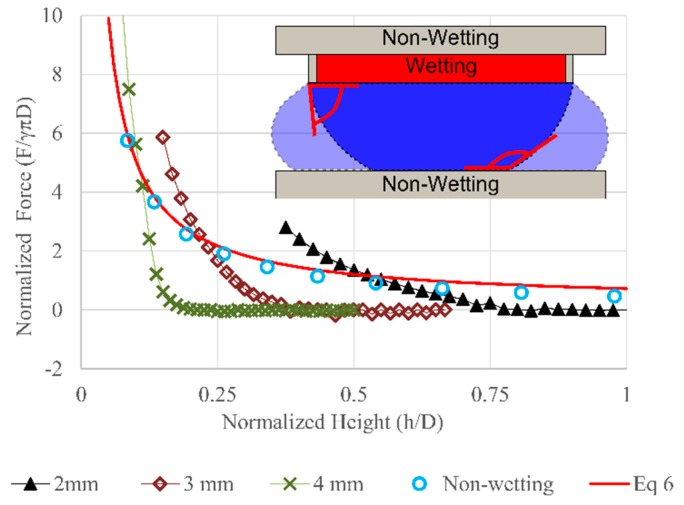
Normalized force vs. normalized height with Type 3 wetting. Results are compared to predictions from Equation (6), the simulation result from Type 1 (symmetric non-wetting, contact angle: 165°, droplet volume 50 µL). The inset shows the wetting region and geometrical constrain on the top plate.

Physical constraints can also be added to the edge of the wetting region to reliably constrain the wetting boundary even beyond 180° (measured relative to the horizontal surface). This could be accomplished using a protruding surface as illustrated in Figure 7. This effect was modeled in Surface Evolver model by constraining the interface so that it could not move beyond the wetting region. The resulting force increases at a much faster rate than Type 1 and 2 as seen in Figure 7.

The Type 3 design can increase the working range of the bearing from 0.1 aspect ratio (*h*/*D*) to at least 0.25. This would be the most favorable design for droplet based bearings. High stiffness and large force could be achieved with larger gap height when the contact line is constrained by both geometry and wetting. As discussed earlier, the viscous drag introduced by the droplet could impose a speed limit on the bearing. By operating at larger height, the drag would be reduced and the maximum speed of the bearing could be increased.

## 4. Discussion

This analysis provides insight into droplet and wetting arrangements that will provide the most accurate fluidic bearing in electrowetting and other applications. To achieve precise translational motion in the *x-y* plane, the relative motion between the top plate and droplet interface on the substrate needs to be minimized. Type 2 and 3 limit the relative motion by constraining the contact line on the moving plate. For a given contact area, smaller droplet volumes would require less gap height to resist the same normal force. Stiffness increases significantly when the top edge is better constrained (Type 3).

For a typical application the size and weight of the plate being carried is fixed. Equation (6) indicates droplet-based bearings favor a large diameter or small gap height. Since the pressure force scales with the diameter squared, larger contact areas can support larger loads. However, a single droplet bearing does not provide much stiffness in rotation about the x and y axis [22] and would add additional uncertainty in plate placement [23]. When multiple droplets support the plate, rotations about the x and y-axes are resisted by the z-stiffness of the droplets. Since two droplets define a line and three droplets define a plane, three droplets would be an appropriate minimum. However, variation in the droplet volume could cause orientation errors which would be reduced by using additional droplets to average out random variation. Additionally, the x and y displacement stiffness is proportional to the droplet circumference which increases when more droplets are used.

Some case studies are used to demonstrate the tradeoffs when designing such bearing. All the calculations are based on Equation (6) (Although the equation is only applicable to Type 1 wetting described above, the calculated force should match closely to Type 2 and 3 at the design point where they intersect the unconstrained droplet force line. While Equation (6) does not predict the stiffness of Type 2 & 3 droplets, it does provide a lower bound for stiffness in these cases.

### 4.1. Design Case 1—Fixed Gap Height

**Figure 8:**
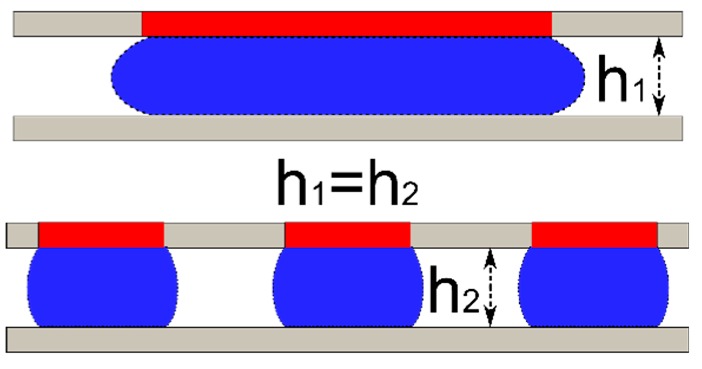
Case study 1: fixed gap height (1 mm) and fixed load (1078 µN). The wetting areas (hydrophilic) are the shaded region. This case compares single vs. multiple droplets for bearing application.

In this first case study (Figure 8), the normal force and the gap height are held constant. The number of droplets and the droplet volume are the variables. The calculated force should be applicable for all types of wetting, but the calculated stiffness is for Type 1 only. From earlier analysis, the stiffness for Type 2 and 3 wetting should be higher than the idealized condition presented here.

For a square glass plate (density 2200 kg/m^3^) with dimension of 10 × 10 × 0.5 mm, the minimal force needed to support the weight of the plate is 1078 µN. Assuming an air ambient and water droplets with surface tension 0.072 N/m, and 110° contact angle on a Cytop coating. At 1 mm gap height, a single 6.8 mm diameter droplet would provide sufficient force to support the plate. However, due to stability issues mentioned above, multiple droplets are preferred. Table 2 shows the impact on the aspect ratio, total droplet area, and the projected length (proportional to the magnitude of an EW actuation force) when using multiple droplets. The supporting force is calculated using Equation (6). This is most accurate for small values of aspect ratio and contact angles near 90° and should be a good approximation for these cases (*h*/*D* < 0.3, *CA* = 110°). As the droplets are parallel springs, the total stiffness is the number of droplets multiplied by the stiffness of a single droplet.

The table above shows the same load could be supported by a number of possible configurations, depending on the design goal. If the normal force per unit area were the main concern, the larger droplet should be used. If the stiffness is the driving parameter, multiple small droplets could achieve higher stiffness. If the actuation force from electrowetting is to be maximized, maximum allowable number of droplet should be used due to the larger project length from multiple droplets.

### 4.2. Design Case 2–Fixed Stiffness and Droplet Diameter

**Figure 9:**
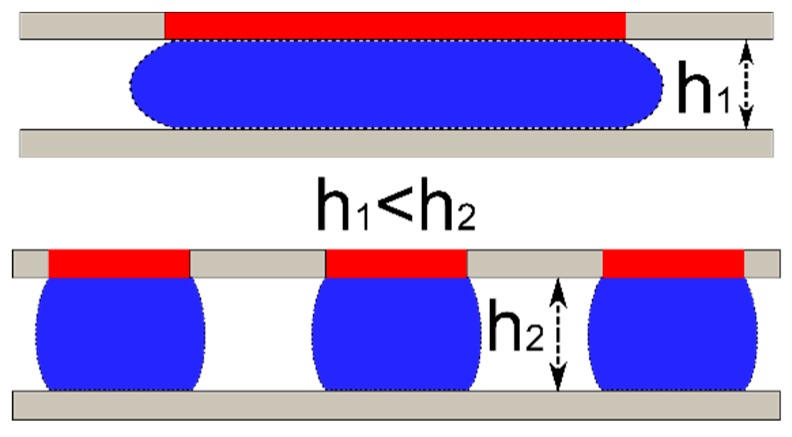
Case study 2: fixed stiffness (162 N/m) and fixed droplet diameter. The wetting areas (hydrophilic) are the shaded region. This case evaluates the impact on droplet volume and gap height when designing for stiffness.

External disturbances such as gravity, shock and vibration will introduce positioning error. When the gap height is small, minor displacement in the vertical direction could cause a large droplet diameter change. This could cause the spacing between the droplets to decrease until they merge. To account for the external disturbance, the stiffness needs to be considered. This case study (Figure 9) uses the same loading (1078 µN) and plate area (100 mm^2^) as above. Instead of fixing the gap height, the bearings are designed for a maximum displacement of the top plate of ±10 µm at 150% overloading. This can be translated to a design requirement of fixed stiffness ($\frac{1.5\times 1078}{10}\;\frac{µN}{µm}=162\frac{N}{m}$). Other parameters such as the surface tension and contact angle remain the same (0.072 N/m, 110°, respectively). The same droplet diameters were used. The gap height is adjusted by varying the volume of the droplet. The same calculation were performed using Equation (6). As seen in Table 3, the aspect ratio needs to be much smaller than the previous case in order to meet the high stiffness requirement. All other outcomes such as the total area required and the actuation force are unaffected due to the fixed wetting diameter. The force approximation is suitable for all wetting conditions. However, due to the higher stiffness of Type 2/3 wetting, the gap height could be much larger for the same stiffness when Type 2/3 wetting is employed.

Another effect of the tradeoffs between design for force and stiffness can be seen in the rapid decrease in the volume of droplets. About one order of magnitude of reduction in volume is required for the increased stiffness. These small volumes could accentuate another error source—droplet volume variation could impact the alignment precision in the x-y plane. This could be offset by increasing the number of droplets to average out random variations which has the benefit of increased gap height for the same force/stiffness values.

### 4.3. Opposing Droplets

**Figure 10:**
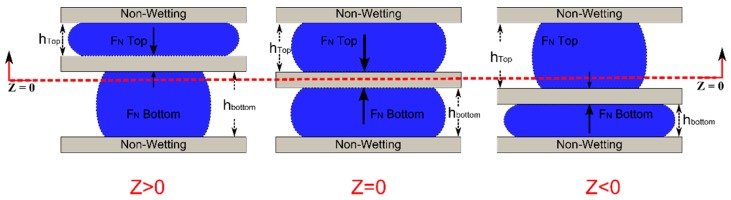
Opposing droplet configuration. Two identical droplets are used to provide support on either side of the center plate. Both the top and bottom plates are fixed. The resulting force and stiffness is symmetrical around the midpoint (*Z* = 0). The left and right illustrations shows the droplet shape when the center plate is displaced around the balance point.

For linear bearing application, the normal force and the stiffness under loading are the primary interests. Higher force enables a larger loading capability and high stiffness would improve the rigidity of the joint. For optimal stiffness and force in the vertical direction, the plate should have both wetting and geometrical constraints. In addition, a preload on the droplet would force the bearing to operate at a higher stiffness range (case 3). In the prior cases, the preload is applied by gravity which makes the plate position sensitive to orientation. Alternatively, we propose a symmetrical droplet bearing which uses opposing droplets to apply a preload to the plate, the resulting force/stiffness could be symmetrical around the middle point which could further increase the reliability of the device—either orientation around the x—y plane is possible. The simplified case with no wetting contrast (Type 1) is analyzed below (Figure 10).

**Figure 11:**
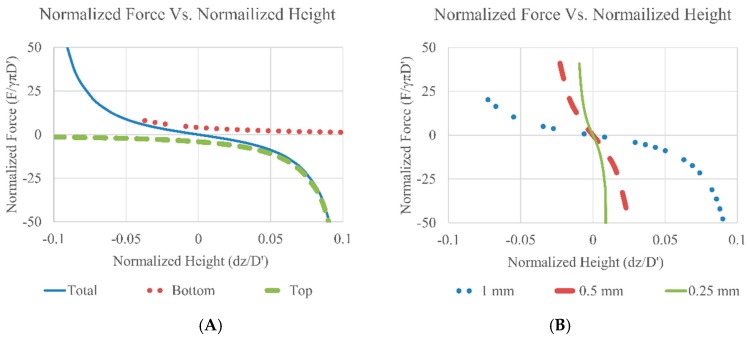
Opposing droplet configuration for optimal stiffness and precise z-location. (**A**) the normalized force from both the top and the bottom droplets are plotted against the normalized height (change in height divided by the nominal droplet diameter, *dh*/*D’*), the equilibrium height is 1 mm for both top and bottom gap. (**B**) Fixed gap heights with changing diameters. The contact angle used in calculation was 165° and the surface tension value used was 0.072 N/m.

To illustrate the force-displacement relation, calculations based on Equation (6) were performed. The force is then normalized by the equilibrium diameter (*D’*) times πγ and the change in height with respect to the middle point (*dz*) was divided by the equilibrium diameter (*D’*). The results are presented in Figure 11. The main assumption is that the weight of the plate is counteracted by the buoyancy force (plate density ≈ ambient fluid density) and all the fluid contacts are non-wetting (Type 1). Implementation of Type 2/3 wetting would significantly increase system stiffness but the trends would be similar. The contact angle values used in the calculation were 165° for both top and bottom. The maximum allowable gap height is fixed at 1, 0.5 and 0.25 mm for both top and bottom gap (without the thickness of the middle plate, *h_Top_* = *h_Bottom_*). The droplet volume was 50 µL each.

As seen in Figure 11A, the top and the bottom droplets exert the same force but in opposite directions around the center (dotted lines), the total force follows a non-linear but symmetrical curve (solid line). When designing the bearing, the droplet contact area could be specified and the height can be tailored to target stiffness. Due to the symmetric force, the location of the plate and/or the stiffness in the *z*-direction could be fine-tuned by using either the volume of the droplet, the wetting pattern or the gap height, to change the preload on the bearing and further improve stiffness as seen in Figure 11B. As the total gap height decreases the droplets are preloaded and the effective stiffness of the droplet bearing increases.

## 5. Conclusions

The normal force of a capillary bridge between two parallel surfaces was analyzed using both Laplace-based calculation and simulation. The force-deflection response of three wetting conditions—uniform wetting, defined wetting region and constrained top-wetting were analyzed using numerical simulation and compared to a simplified closed form approximation. This approximation is shown to provide useful reference information for predicting key aspects of each wetting type. For all cases, smaller gaps create higher stiffness and larger forces. The peak force and stiffness are achieved with geometrical constraints on the top plate that enable effective contact angles above 180° at the edge of the wetting boundary.

For ultimate precision, an opposing droplet configuration was proposed. Simplified force analysis showed a symmetrical stiffness response due to the preloading effect. The stiffness could be further fine-tuned by changing the total gap height. For linear droplet bearing actuated by electrowetting, a series of design cases were presented to demonstrate the advantage of using multiple droplets to support the same load. The larger actuation force capability of multiple droplets could improve the speed of the actuator. When designing such actuators, the total area and the gap height should be the driving parameters to meet required load and stiffness performance levels.

## Figures and Tables

**Table 1:** Simulation parameters used.

Droplet volume	5–50 µL (5 µL increments)
Contact angle (both top and bottom)	165°, 120°, 110°
Gap height	0.5–3.0 mm

**Table 2:** Case study of droplet(s) supporting a fixed load at constant gap height, the contact angle is 110° top and bottom. The total supporting force is 1078 µN and the gap height is 1 mm.

# of Droplets	Volume of Each Droplet (µL)	Aspect Ratio (h/D)	Diameter of the Drop (mm)	Total Stiffness (N/m)	Minimum Total Area Required (mm^2^)	Total Projected Length (mm)
1	36.3	0.147	6.8	1.8067	36.3	6.8
3	16.6	0.217	4.6	2.48031	49.9	13.8
5	12.6	0.25	4	3.12578	62.8	20
7	10.8	0.27	3.7	3.7443	75.3	25.9

**Table 3:** Case study of droplet(s) with constant stiffness (162 N/m), the contact angle is 110° top and bottom. The diameter of the top wetting region is the same as in case study 1.

# of Droplets	Volume of Each Droplet (µL)	Aspect Ratio (h/D)	Diameter of the Drop (mm)	Height of the Gap (mm)	Minimum Total Area Required (mm^2^)	Total Projected Length (mm)
1	3.8	0.015452	6.8	0.105	36.3	6.8
3	2.1	0.026764	4.6	0.123	49.9	13.8
5	1.7	0.034553	4	0.138	62.8	20
7	1.6	0.040883	3.7	0.151	75.3	25.9
